# Stress-associated changes in salivary microRNAs can be detected in response to the Trier Social Stress Test: An exploratory study

**DOI:** 10.1038/s41598-018-25554-x

**Published:** 2018-05-08

**Authors:** Conrad Wiegand, Peter Heusser, Claudia Klinger, Dirk Cysarz, Arndt Büssing, Thomas Ostermann, Andreas Savelsbergh

**Affiliations:** 1 Senior Professorship for Medical Anthropology, Institute of Integrative Medicine, Witten/Herdecke University, Herdecke, Germany; 2 Chair for Biochemistry and Molecular Medicine, Division of Functional Genomics, Witten/Herdecke University, Witten, Germany; 3 Center for Biomedical Education and Research (ZBAF), Witten/Herdecke University, Witten, Germany; 4 Integrated Curriculum for Anthroposophic Medicine, Witten/Herdecke University, Herdecke, Germany; 5 Professorship Quality of Life, Spirituality and Coping, Institute of Integrative Medicine, Witten/Herdecke University, Herdecke, Germany; 6 Chair of Research Methodology and Statistics in Psychology, Department of Psychology and Psychotherapy, Witten/Herdecke University, Witten, Germany

## Abstract

Stress is an important co-factor for the genesis and maintenance of many diseases and is known to have an effect on gene expression via epigenetic regulation. MicroRNAs (miRNAs) appear to function as one of the key factors of this regulation. This is the first study to investigate the response of 11 stress-associated miRNAs in human saliva - as a non-invasive source - in an experimental condition of acute psychological stress, and also their correlation with established psychological (subjective stress perception), physiological (heart rate and heart rate variability) and biochemical stress parameters (salivary cortisol and alpha-amylase). 24 healthy participants between 20 and 35 years of age were investigated, using the Trier Social Stress Test (TSST) to induce acute psychological stress. Stress-associated changes were significant for miR-20b, -21 and 26b, and changes in miR-16 and -134 were close to significance, recommending further research on these miRNAs in the context of stress reactions. Significant correlations with alpha-amylase suggest their integration in sympathetic stress regulation processes. Additionally, our results demonstrate the TSST as a reliable tool for studying salivary miRNAs as non-invasive indicators of epigenetic processes in acute psychological stress reactions.

## Introduction

Stress is an important co-factor for the genesis and maintenance of acute and chronic diseases and thus of epidemiological and health-economic relevance^1^. Stress can occur in different forms and intensities. Focusing on psychological stress, it can be defined as a psychological state that occurs when “an individual perceives that environmental demands tax or exceed its adaptive capacity”^2,3^. Furthermore, the subjective perception of (psychological) stress is individual and depends on individual coping capacities^4^. It leads to several responsive processes on the physiological, cellular and molecular levels of the organism^5^. Some physiological reactions can be reliably elicited through standardized experimental social stress tests such as the “Trier Social Stress Test” (TSST)^6^ and are generally accepted as biological markers for psychological stress. This applies to salivary cortisol levels as a marker for the activation of the hypothalamic-pituitary-adrenal (HPA) axis^7^ and to the activation of salivary alpha-amylase as a surrogate marker for sympathetic activation^8^.

Many stress reactions are already well-investigated and certain pathways of the involved processes are explored in detail. However, specific regulations still have to be clarified. Important candidates for stress-related regulation are microRNAs (miRNAs). miRNAs are a group of small, non-coding RNAs, about 22–23 nucleotides in length^9,10^. miRNAs generally play an important role in gene-expression by post-transcriptional gene-regulation, via targeting messengerRNAs (mRNAs), inducing their degradation, cleavage or translational repression^9–11^. They have been found to be involved in various stress-reactive processes and therefore are suggested to exert a regulative function in them^5^.

miRNAs can be found not only intracellularly, but also outside of cells in several body fluids such as in human whole blood^12^, serum^13^, plasma^14^ and saliva^15^. Because of their stable presence in those body fluids^14^, which in saliva is due to their packaging in exosomes^16^, miRNAs have been suggested to be suitable as potential biological markers for various processes of the organism. As such they have already been described for several diseases in humans, especially in several forms of cancer such as in oral squamous cell carcinoma^17^, esophageal cancer^18^, non-small cell lung cancer^19^, and for the detection of resectable pancreatic cancer^20^ as well as for autoimmune diseases such as Sjögren’s syndrome^15,21^. Salivary miRNAs have not been described as potential biological markers for psychological stress reactions so far.

miRNAs obtained from human blood have already been used as biological markers in the assessment of psychological stress, either under persisting chronic mental stress conditions^12,22^ or in an experimental setting of acute psychological stress^23,24^. However, for reasons of ethics and practicality, especially in an experimental setting of an acute psychological stress test, theoretically the best method for obtaining biomarkers would be the least invasive one, namely from saliva^5^.

For this reason we analyzed recent literature to identify promising miRNAs for the use as salivary indicators of epigenetic processes in an acute standardized psychosocial stress test^5^. We found 11 candidates that seemed eligible for this study design: miR-16; -20b; -26b; -29a; -126; -144; -144*^12,22^; -134; -183^25^; -10a; -21^23^ (Table 1).

**Table 1:** microRNA Designation.

miR-10a	hsa-miR-10a-5p
miR-16	hsa-miR-16-5p
miR-20b	hsa-miR-20b-5p
miR-21	hsa-miR-21-5p
miR-26b	hsa-miR-26b-5p
miR-29a	hsa-miR-29a-3p
miR-126	hsa-miR-126-3p
miR-134	hsa-miR-134-5p
miR-144	hsa-miR-144-3p
miR-144*	hsa-miR-144-5p
miR-183	hsa-miR-183-5p

Based on these findings we conceived the present exploratory study to explore if these miRNAs obtained from saliva react to a standardized stress exposure and whether the TSST is an appropriate method for eliciting such stress-associated changes of salivary miRNAs. Hence, we aimed at two innovations; (a) to investigate these stress-associated miRNAs in human saliva and (b) to investigate them in the setting of the TSST as a standardized acute psychosocial stress test.

To this aim we investigated the expression levels of these 11 miRNAs in the exosomal fraction of human saliva at different time-points before, during and after undertaking the TSST. Also, as the specific functions and roles of these stress-associated miRNAs in the context of overall-stress response often remain unclear, we analyzed statistical correlations of their course (1) with the course of subjectively perceived stress, (2) with salivary cortisol as an indicator of the HPA-axis^7^, as well as salivary alpha-amylase, and (3) with heart rate (HR) and heart rate variability parameters (HRV) assessed in parallel as indicators of sympathetic tone activation during stress exposure^26–28^. This might contribute to understand the functional integration of these miRNAs in the overall stress-response.

**Figure 1:**
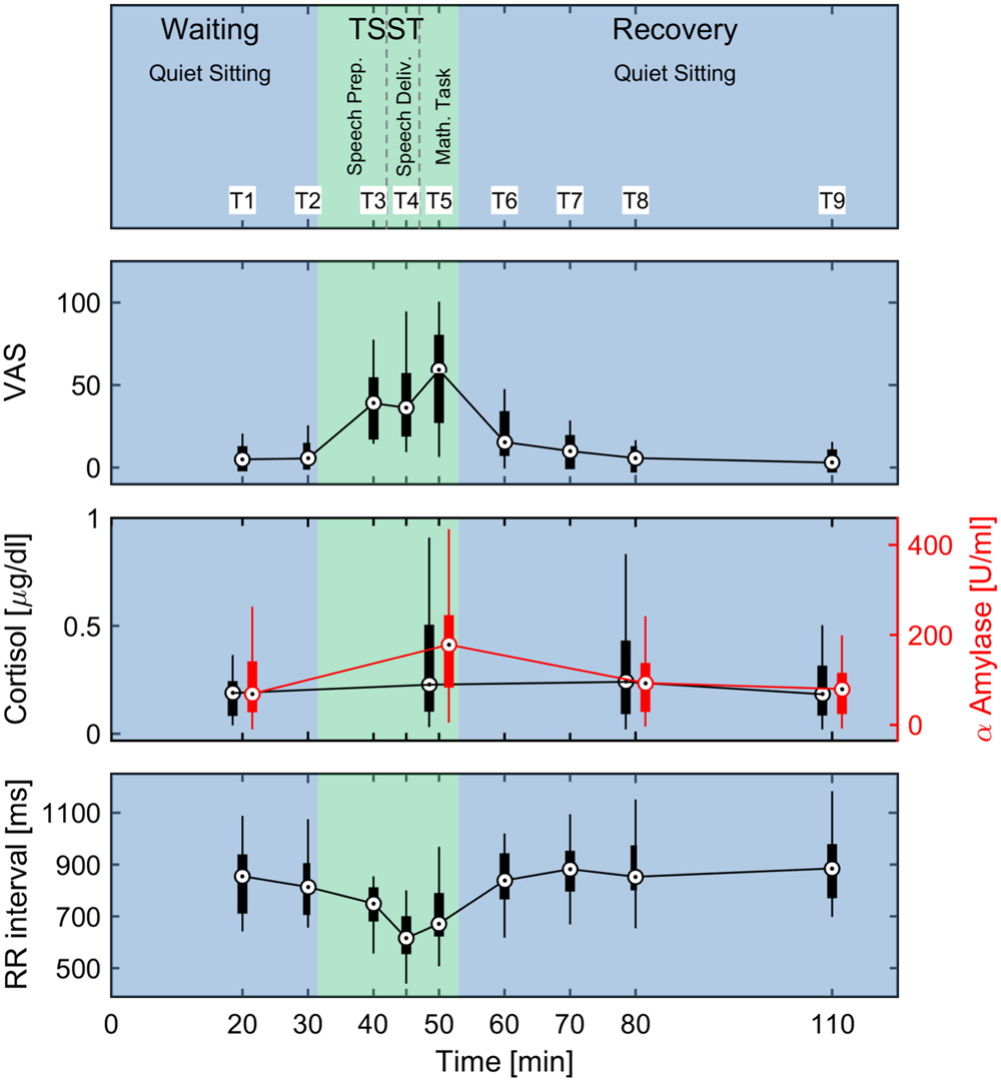
Experimental design, course of established stress parameters. The central circle represents the median; the boxplots represent the distribution from 25%- to 75%-percentile. The Whiskers represent remaining data distribution from minimum to maximum.

24 healthy participants between 18 and 30 years old, 12 female and 12 male, were investigated in the TSST, using a modified version of an established TSSST protocol^29^ (Fig. 1).

We hypothesized that the responsive miRNA expression levels would change during the stress test and then normalize steadily to baseline until about one hour later. We also hypothesized that some miRNA expression levels would correlate with the expression of the established psychological, physiological and biochemical stress parameters mentioned above.

## Aim

The aim of the study was to explore whether stress-related salivary miRNAs (in the exosomal fraction) react to a standardized experimental acute psychosocial stress exposure and correlate with standard psychological, physiological and biochemical stress parameters. Furthermore, we investigated whether the TSST is a suitable tool for the investigation of changes in stress-associated salivary miRNAs. As this study is explorative in character, it generally aims to provide a first experimental ground on which further investigations to identify salivary miRNAs as stress-associated, non-invasive biomarkers can be built upon. It does not aim to investigate this in the first place.

### Primary research questions

1. Can stress-related miRNAs detected in the exosomal fraction of human saliva be significantly altered in response to an acute standardized social stress exposure?
2. Is the TSST an appropriate tool for eliciting such a response of stress-associated miRNAs in human saliva?

### Secondary research questions

1. Do responding miRNAs correlate with psychologically perceived stress?
2. Do responding miRNAs correlate with stress-associated modifications of heart rate and heart rate variability as physiological stress parameters?
3. Do responding miRNAs correlate with stress-associated salivary alpha-Amylase and salivary Cortisol as biochemical stress parameters?

## Results

### TSST – standardized stress parameters reactivity

#### Visual Analogue Scale (VAS)

Subjectively perceived stress as quantified by a VAS, ranging from 0 (feeling no stress at all) to 100 (feeling maximally stressed), showed significant changes across the procedure (p_Skillings-Mack_ < 0.001). The median VAS increased from the waiting period T1 (5) to the TSST test-period, peaking at the end of the TSST at T5 (59) directly after the arithmetic task, and then decreased continuously from T5 to T9 (end of recovery period) back to the level baseline (Table 2/Fig. 1). As the times T3 (speech preparation), T4 (speech delivery) and T5 (arithmetic task) were within the TSST (Fig. 1), the differences between them should be seen as the individual stress perception corresponding to the respective tasks.

**Table 2:** Results of visual analogue scale and heart rate/heart rate variability.

	T1	T2	T3	T4	T5	T6	T7	T8	T9
VAS^#^	5 1–10	6 2–12 T1	39 20–52 T1, T2	36 22–57 T1, T2, T3	59 30–78 T1, T2, T4	16 10–31 T1,T2,T3, T4,T5	10 2–17 T1,T2,T3, T4,T5,T6	6 0–10 T2,T3,T4, T5,T6,T7	3 0–8 T2,T3,T4, T5,T6,T7
RR intervall (ms)*	855 720–930	813 724–887	750 699–793 T1,T2	616 573–682 T1,T2	672 641–771 T1,T2,T3, T4	839 785–924 T1,T2,T3, T4,T5	882 815–934 T1,T2,T3, T4,T5,T6	853 819–955 T1,T2,T3, T4,T5,T6	885 789–960 T1,T2,T3, T4,T5,T6
SDNN (ms)*	66 52–95	76 68–89	72 51–94	61 48–75 T1,T2,T3	58 45–72 T1,T2,T3	80 64–104 T4,T5	72 62–97 T4,T5	81 62–109 T4,T5	73 59–100 T1,T4,T5
RMSSD (ms)*	37 30–49	35 29–48	33 27–49	21 16–28 T1,T2	25 16–35 T1,T2,T3	48 34–62 T1,T2,T3 T4,T5	44 35–55 T1,T2,T3 T4,T5	46 32–58 T1,T2,T3 T4,T5	40 30–56 T1,T2,T3 T4,T5,T6

Median and IQR of parameters assessed at 9 times.

^#^p_Skillings-Mack_ < 0.001.

*p_Friedman_ < 0.001.

The first row lists median, the second row lists the 25%- and 75%- percentile (IQR). The lowermost row of each parameter lists significant differences to other time points (p < 0.05).

#### Heart Rate Variability (HRV)

The increase in perceived stress during the TSST was accompanied by physiological stress as assessed by parameters of HRV. Stress decreased the median RR interval (T4: 616 ms; Table 2, Fig. 1), i.e., the heart rate was highest (p_Friedman_ < 0.001). The waiting period before the TSST showed a longer median RR interval (T1: 855 ms) and the recovery period after TSST showed the longest median RR interval (T9: 885 ms), i.e., heart rates were lower before and after the TSST (Table 2, Fig. 1). The decrease of the median RR interval during the TSST was accompanied by a decrease of ‘Standard deviation of all normal-to-normal intervals’ (SDNN) and ‘Root of the mean squared difference of successive RR intervals’ (RMSSD), indicating a lower HRV during the TSST (p_Friedman_ < 0.001; Table 2). The median SDNN was lowest during arithmetic task (T5;58 ms), whereas the median RMSSD was lowest during the speech delivery (T4;21 ms). SDNN and RMSSD were higher at rest before the TSST (T1, SDNN: 66 ms, RMSSD: 37 ms) and even slightly higher at the end of the recovery procedure after the TSST (T9, SDNN: 73 ms, RMSSD: 40 ms). These results indicate that rest after the TSST was slightly better than rest before the TSST.

#### Cortisol and alpha-Amylase

Salivary alpha-amylase activation (p_Friedman_ < 0.001) and salivary cortisol levels (p_Friedman_ < 0.05) also showed changes across the procedure (Table 3, Fig. 1). The median level of alpha-amylase activation, i.e., the concentration of salivary alpha-amylase increased from waiting period before the stress test to the stress test (T1: 69 U/ml, T5: 178 U/ml). The recovery period after the stress test showed the same median level as rest before the stress test (T9: 80 U/ml).

**Table 3:** Results of salivary cortisol, alpha-amylase and miRNAs.

	T1	T5	T8	T9	d
Cortisol (μg/dl)**	0.19 0.11–0.22	0.23 0.13–0.48 T1	0.24 0.12–0.41	0.18 0.11–0.29 T5, T8	0.8216
α-Amylase (U/ml)***	69 39–130	178 94–233 T1, T8, T9	93 40–127	80 35–105	2.5182
miRNA16 (ΔCT)*	−0.37 −1.14–0.38	−0.75 −1.83–0.53	−0.17 −0.66–0.53	0.00 −0.49–0.55 T5	0.6796
miRNA20b (ΔCT)^##^	2.74 1.26–5.22	2.11 1.40–2.53 T1	2.74 1.63–4.10	1.85 0.51–3.64 T1	0.7103
miRNA21 (ΔCT)**	−0.82 −1.77–0.74	−0.39 −1.45–0.36	0.57 −0.55–1.08 T1, T5	0.67 −1.04–1.64 T1, T5	0.8563
miRNA26b (ΔCT)^###^	0.85 −0.77–2.08	0.11 −0.45–1.44	1.46 0.26–2.32 T5	1.08 −0.07–2.59	1.1236
miRNA29a (ΔCT)	−0.37 −1.59–0.62	−0.86 −1.63–0.31	−0.50 −1.45–1.13	−0.08 −1.59–1.41	0.2768
miRNA134 (ΔCT)^#^	1.79 1.25–3.11	1.47 0.40–2.34	2.62 1.41–3.52 T5	2.80 0.31–3.23	0.6622

Median and IQR of parameters assessed at times T1, T5, T8, T9 and ‘Cohen d’ effect size.

*p_Friedman_ = 0.059; **p_Friedman_ < 0.05; ***p_Friedman_ < 0.001.

^#^p_Skillings-Mack_ = 0.068; ^##^p_Skillings-Mack_ < 0.05; ^###^p_Skillings-Mack_ < 0.001.

The first row lists median, the second row lists the 25%- and 75%- percentile (IQR). The lowermost row of each parameter lists significant differences to other time points (p < 0.05). (Remark: ΔCT-values are inverse to miRNA-concentrations in the samples: low ΔCT-values indicate high miRNA concentrations/high ΔCT-values indicate low miRNA concentrations).

The median concentration of salivary cortisol was lowest during the waiting period and recovery period, before and after the TSST (T1: 0.19 µg/dl, T9: 0.18 µg/dl; Table 3, Fig. 1). The cortisol concentration also showed changes over time, but the stress test did not induce a clear peak of salivary cortisol concentration. Instead, T5 and T8 showed similar cortisol concentrations (T5: 0.23 µg/dl, T8: 0.24 µg/dl; Table 3, Fig. 1). Nevertheless, only the cortisol level at T5 differed from the levels at T1 and T9 indicating that cortisol was systematically increased only at T5. The cortisol level at T8 differed only from T9 indicating a slight decrease towards baseline level.

### miRNA-expression responses on TSST as epigenetic parameters

In total, 11 salivary miRNAs (miR-10a; -16; -20b; -21; -26b; -29a; -126; -134; -144; -144*; -183) were tested. The miRNAs miR-10a; -126; -144; -144* and -183 could not be detected reliably in the saliva samples collected. However, 6 miRNAs (miR-16; -20b; -21; -26b; -29a and -134) could be detected reliably and changes in their concentration levels could be analyzed across the test procedure.

Of these, three miRNAs showed significant changes across the test procedure: miR-20b (p_Skillings-Mack_ < 0,05), miR-21 (p_Friedman_ < 0,05) and miR-26b (p_Skillings-Mack_ > 0,001). Changes in two miRNAs were not significant but showed trends close to significance: miR-16 (p_Friedman_ = 0,059) and miR-134 (p_Skillings-Mack_ = 0,068).

Changes in miR-29a did not show significance, neither a trend close to significance, but were significantly correlated with changes in several other parameters assessed in this study.

**Figure 2:**
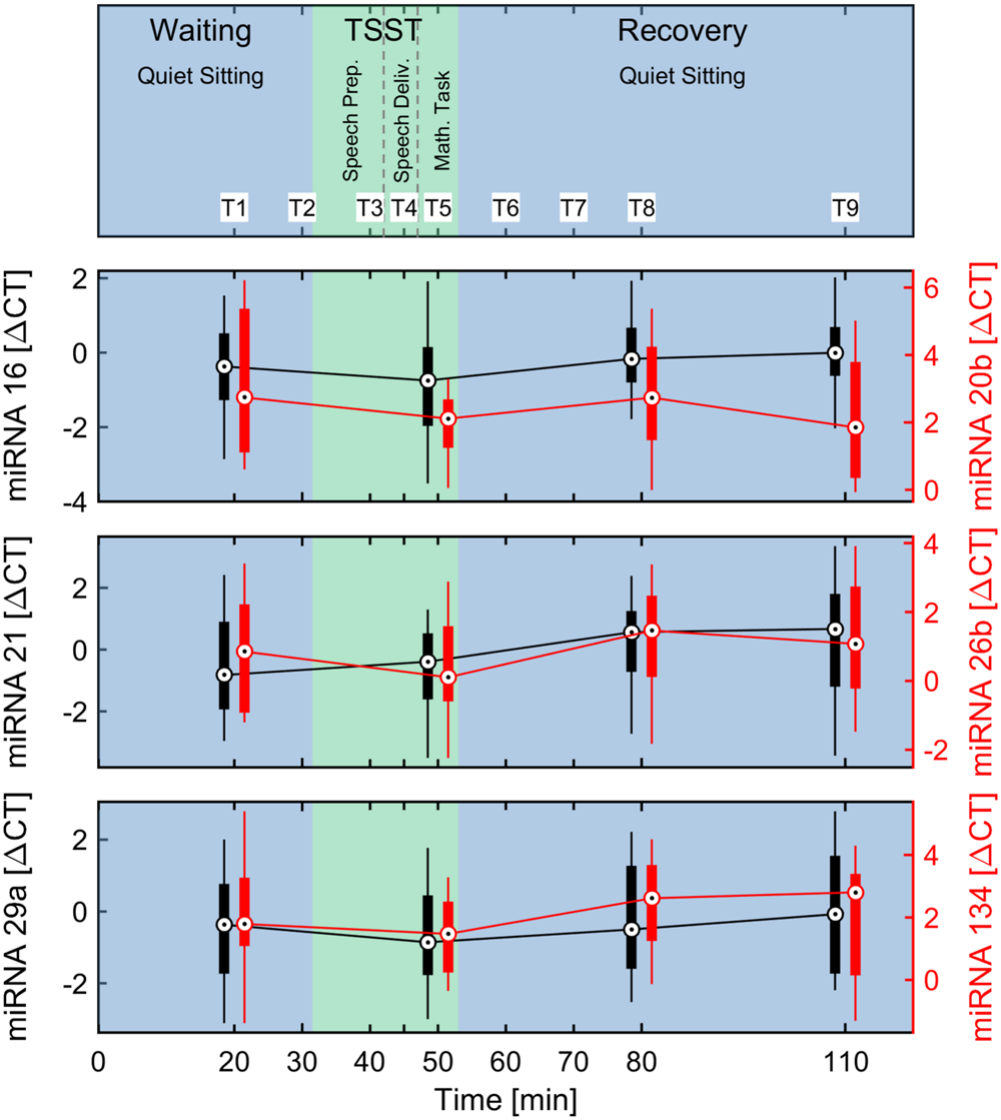
**C**ourse of salivary miRNAs. The central circle represents the median; the boxplots represent the distribution from 25%- to 75%-percentile. The Whiskers represent remaining data distribution from minimum to maximum.

The median expression of miR-21 (p_Friedman_ < 0.05) was higher at T1 and T5 (T1: Δ‘cycle threshold’ (CT) = −0.82, T5: ΔCT = −0.39) compared to times T8 and T9 (T8: ΔCT = 0.57, T9: ΔCT = 0.67). Overall, its expression decreased continuously from T1 to T9, showing the strongest decrease directly after the TSST from T5 to T8 (Table 3, Fig. 2). Median miR-26b expression was highest during T5 (ΔCT = 0.11), increasing from T1 (ΔCT = 0.85) and decreased at T8 (ΔCT = 1.46; Table 3, Fig. 2). The median expression of miR-20b (p_Skillings-Mack_ < 0.05) showed two peaks: increasing from T1 (ΔCT = 2.74) to T5 (ΔCT = 2.11), then decreasing again to baseline at T8 (ΔCT = 2.74), showing a second and highest peak at T9 (ΔCT = 1.85; Table 3, Fig. 2).

The expression levels of miR-16 and miR-134 showed a non-significant tendency to change across the procedure. For miR-16 the changes of were close to the significance level (p_Friedman_ = 0.059) and, hence, the median expression level at T5 (ΔCT = −0.75) was likely higher compared to T9 (ΔCT = 0.00). Changes of miR-134 were also close to the significance level (p_Skillings-Mack_ = 0.068) and, hence, at times T1 and T5 the median expression level was higher and the level decreased at times T8 and T9. The post-hoc test indicated a decrease between T5 and T8 (T5: ΔCT = 1.47, T8: ΔCT = 2.62).

The post-hoc calculation of effect sizes η^2^, performed to test whether the a priori estimated effect sizes were justified, demonstrated large effects (d > 0.8) for miR-21 (n _post-hoc_ = 17, d = 0.8563) and miR-26b (n _post-hoc_ = 11, d = 1.1236) as well as a medium effect (d = 0.6–0.8) for miR-20b (n _post-hoc_ = 24, d = 0.7103; Table 3). Medium effects were also demonstrated for miR-16 (n _post-hoc_ = 26, d = 0.6796) and miR-134 (n _post-hoc_ = 27, d = 0,6622).

### Correlations among parameters

As already mentioned in the introduction, statistical correlations were calculated to obtain information about possible functional relations between the miRNAs and the other stress- parameters in the context of the overall stress reaction.

**Figure 3:**
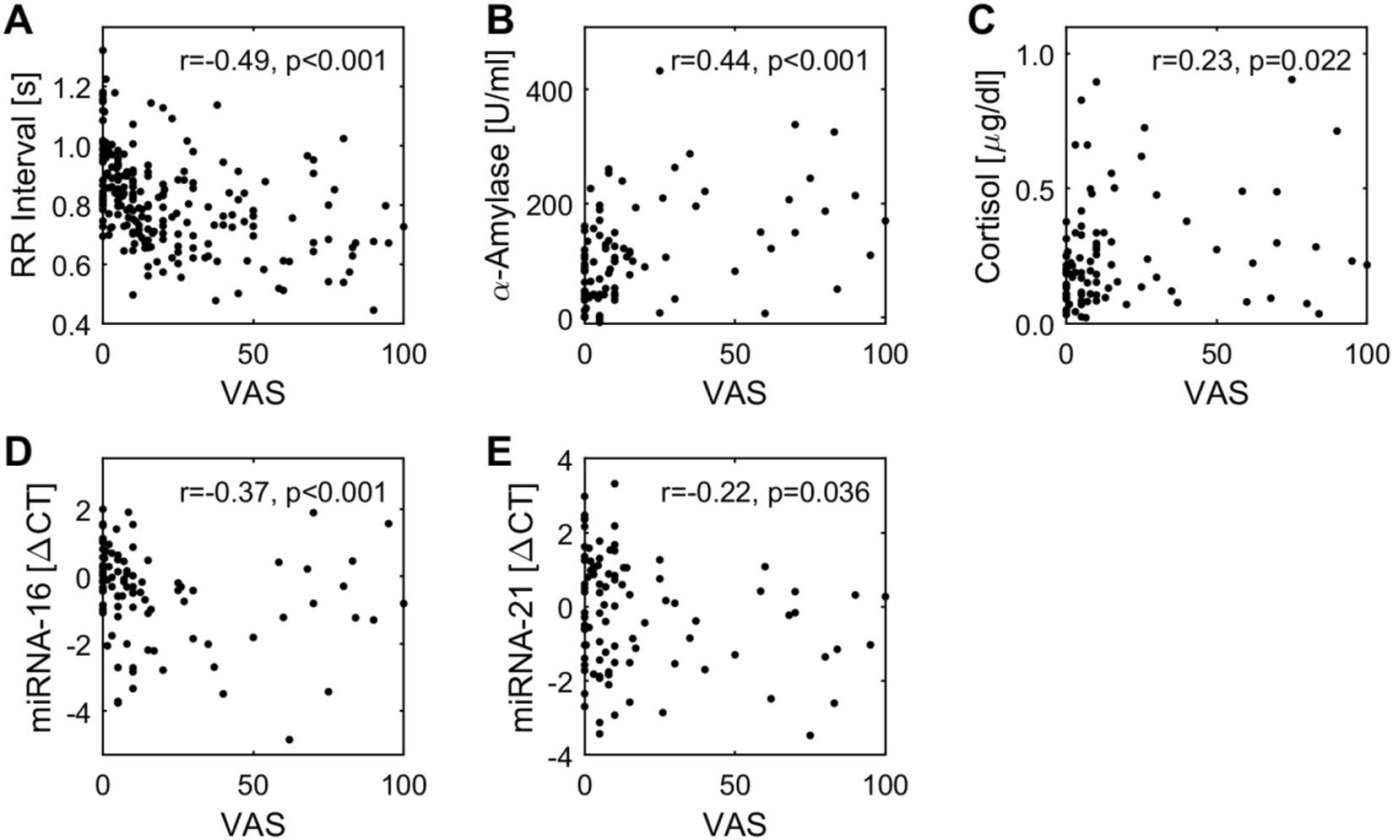
Correlations between VAS and other parameters. All correlations were calculated using Spearman rank correlation procedure. p < 0.05 as considered significant, p < 0.10 was considered showing tendency towards significance.

Perceived stress (VAS) showed correlations with several parameters. VAS was moderately negative correlated with the average RR interval (r = −0.49, p < 0.001; Fig. 3A), i.e., the higher VAS the lower the average RR interval. At the same time stress perception correlated moderately positive with alpha-amylase (r = 0.44, p < 0.001; Fig. 3B) and weakly with cortisol concentrations (r = 0.23, p < 0.05; Fig. 3C) and, i.e., the higher the stress perception, the higher cortisol or alpha-amylase. Alpha-amylase and cortisol concentration were moderately interrelated (r = 0.42, p < 0.001).

**Figure 4:**
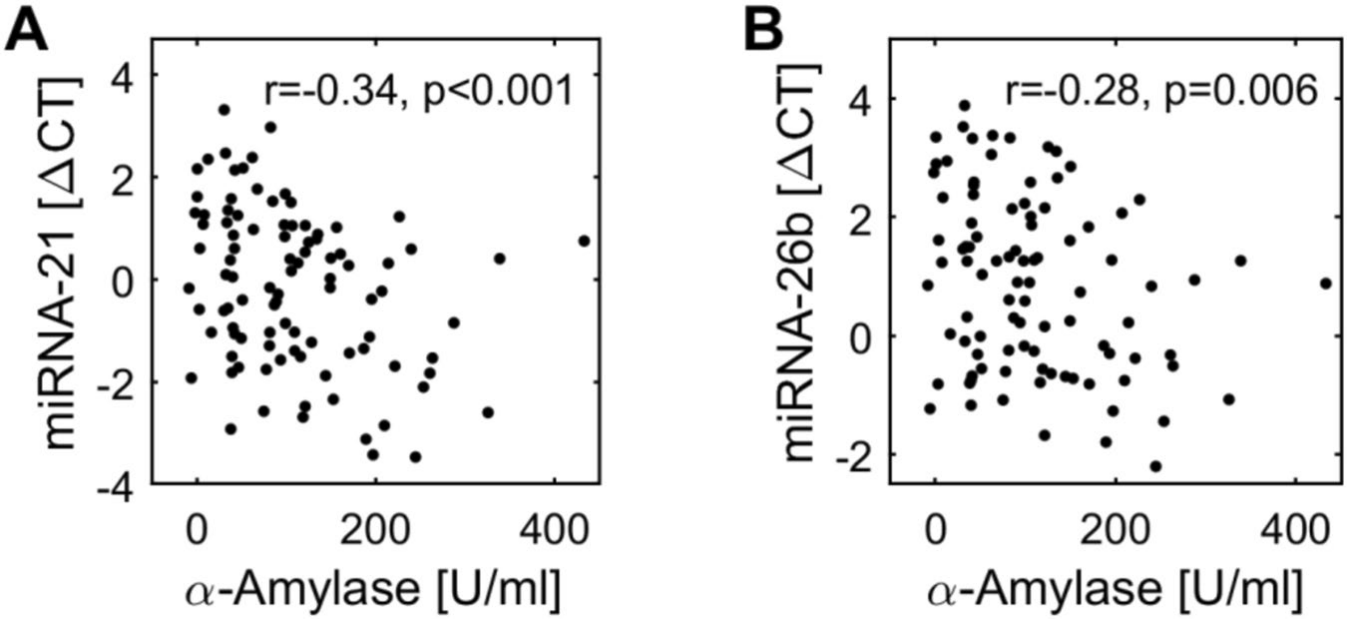
Correlations between alpha-amylase and miR-21/miR-26b. All correlations were calculated using Spearman rank correlation procedure. p < 0.05 as considered significant, p < 0.10 was considered showing tendency towards significance.

With respect to the epigenetic parameters, VAS showed weak to moderate negative correlations to the ΔCT-values of miR-16 (r = −0.37, p < 0.001; Fig. 3D) and miR-21 (r = −0.22, p < 0.05; Fig. 3E). This corresponds to a positive correlation with miRNA-concentrations, as lower ΔCT-values indicate higher miRNA concentrations (see Methods section). The concentration of alpha-amylase was negatively correlated weakly to moderately with the ΔCT-values of miR-21 (r = −0.34, p < 0.001; Fig. 4A) and miR-26b (r = −0.28, p < 0.01; Fig. 4B), i.e., the higher alpha-amylase, the higher the miRNA-concentration or the epigenetic reaction, respectively.

**Figure 5:**
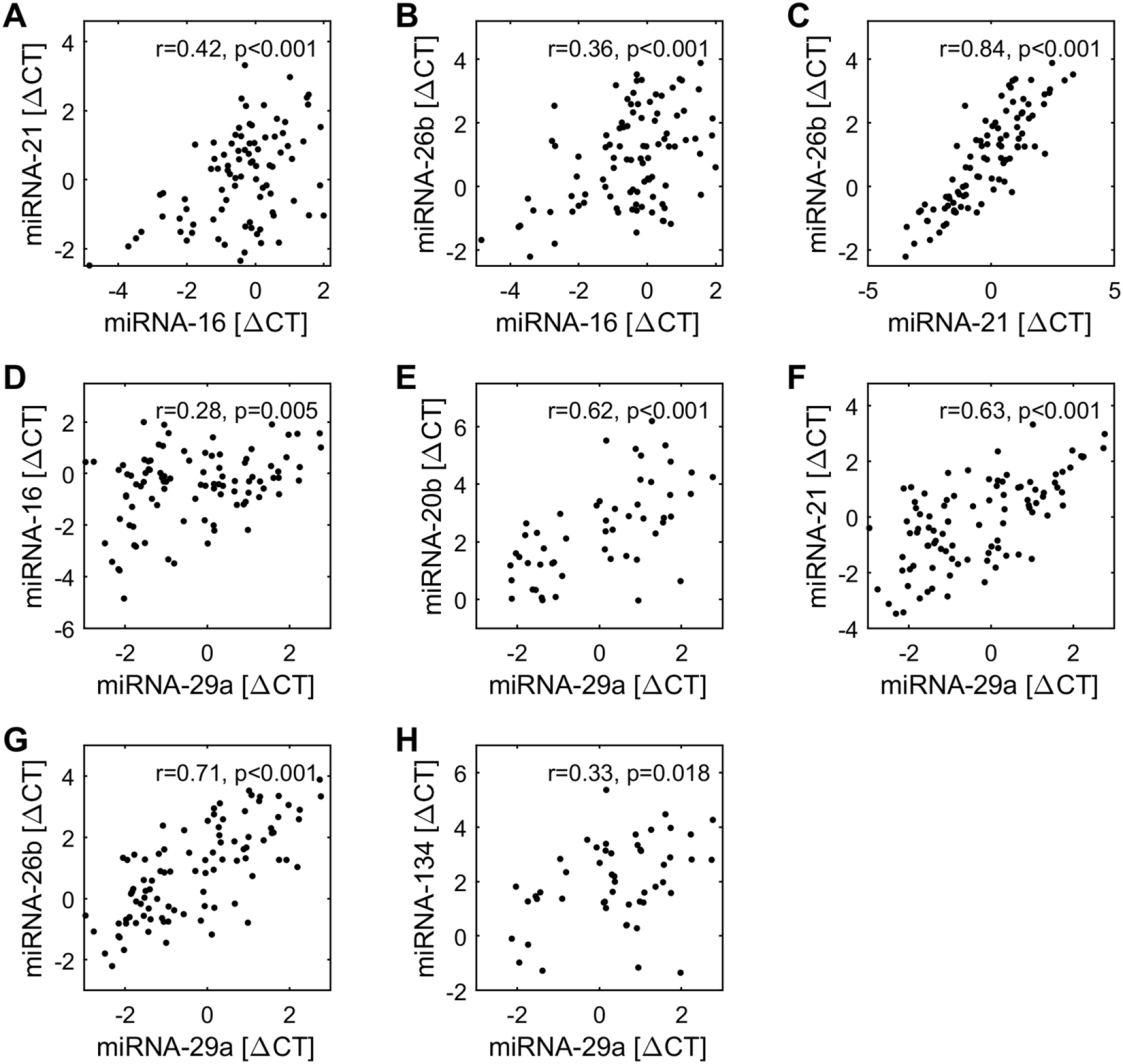
Correlations among miRNAs. All correlations were calculated using Spearman rank correlation procedure. p < 0.05 as considered significant, p < 0.10 was considered showing tendency towards significance.

Furthermore, significant correlations among the epigenetic parameters were found. miR-21 and miR-26b showed a strong correlation (r = 0.84, p < 0.001; Fig. 5C), i.e., if the ΔCT-values of miR-21 were high, it was very likely that the same was true for miR-26b. The ΔCT-values of miR-21 were also moderately positive correlated with miR-16 (r = 0.42, p < 0.001; Fig. 5A). Furthermore, miR-26b and miR-16 were positively interrelated, too (r = 0.36, p < 0.001; Fig. 5B).

Interestingly, miR-29a showed significant correlations to all of the reliably detectable miRNAs. Even though changes in the concentration of miR-29a across the procedure were not significant (p = 0,717). The ΔCT-values of miR-29a strongly correlate with miR-20b (r = 0.62, p < 0.001; Fig. 5E), miR-21 (r = 0.63, p < 0.001; Fig. 5F) and miR-26b (r = 0.71, p < 0.001; Fig. 5G), and weakly to moderately with miR-16 (r = 0.28, p < 0.01; Fig. 5D) and miR-134 (r = 0.33, p < 0.05; Fig. 5H). No other significant correlations for miR-134 were found.

## Discussion

As the results of the subjective stress perception (VAS) indicate a clear acute stress-induction in the participants responding to the TSST, the results of the HRV parameters indicate an increased activation of the sympathetic nervous system.

The salivary concentration levels of cortisol and alpha-amylase also showed stress reactive changes compatible with these psychological and physiological stress parameters. Such measurements of the subjective stress perception as well as the reaction of heart rate peaking during and salivary alpha-amylase peaking at the end of the TSST have already been described in current literature^8,30^. Gordis *et al*. described the concentration salivary cortisol to peak 10 minutes after the concentration salivary alpha-amylase responding to the TSST. Such time differences could not be detected in our study, due to an interval of 30 minutes between T5 (directly after the TSST) and T8. However, as we found almost similar courses for both, cortisol and amylase showing a plateau from T5 to T8, such peaks in between T5 and T8 can be expected regarding the findings of Gordis *et al*.

The increase of salivary alpha-amylase confirms the increased activation of the sympathetic nervous system, too. Furthermore, the changes in salivary cortisol concentrations indicate an increased activation of the HPA-axis in response to the TSST. Lucassen *et al*. have already described such a temporally delayed response of the HPA-axis compared to the sympathetic activation^31^.

Therefore, these results demonstrate that the TSST leads to a significant induction of subjectively perceived stress and a concomitant physiological stress response, as described in recent literature.

### Putative relevance of identified miRNAs

As an answer to our primary research question, we found 6 of the 11 miRNAs tested (miR-16; -20b; -21; -26b; -29a and -134) to be reliably detectable in the exosomal fraction of our subjects’ saliva. Of these, miR-20b, -21, and -26b showed significant changes in response to the TSST, while the changes in miR-16 and-134 were only close to significance (Table 3, Fig. 2). Furthermore, changes in the concentration of miR-29a correlate significantly with changes in all of these 5 miRNAs, even though its own changes were not significant. This suggests an associated expression of miR-29a with these miRNAs or even a common preceding regulatory process.

Effect size calculations of miRNAs were in accordance with the a priori estimated effects, providing large effects for miR-21 and miR-26b as well a medium effect for miR-20b. Hence, the sample size of n = 24 seems to be appropriate for the present study design and research questions.

Our analysis of current published knowledge on the miRNAs tested in our study revealed numerous findings about miR-21. Particularly, a miR-21-overexpression in several types of cancer has been described^32^. As an oncogene, it is involved in the regulation of apoptosis^33^ and promotes tumor genesis through its regulation of cellular Reactive Oxygen Species (ROS) levels by directly inhibiting Superoxide dismutase 2 (SOD2), one of the key molecules of cellular coping mechanisms^34^. ROS are the main factors for the induction of the oxidative stress response as one of the pathways of cellular stress response^35^. Therefore, miR-21 might also be involved in the cellular stress response coping with oxidative stress, which in turn also appears to be a result of perceived psychological stress^5,36^. Furthermore, adrenaline has been found to directly interact with miR-21, i.e. by a down-regulation of its expression levels in human bone marrow-derived stem cells and the suppression of osteogenic differentiation^32^.

Gidron *et al*. have demonstrated findings comparable to those we found in salivary miR-21, albeit in a response to psychological stress in whole blood: They report a decrease of miR-21 concentration in whole blood of healthy humans under subjectively perceived psychological stress as opposed to non-stressed samples of the same subjects^37^. However, the time lag between both time points was weeks, and university exams were used as stressor, indicating a more chronic mental stress situation, while in our study we focused on acute stress-dependent changes. On the other hand, Beech and coworkers reported an increase of miR-21 concentration in human whole blood one hour after the induction of acute psychological stress through an imaginary mental stress test^23^. These results appear contradictory to the results we found; a continuous decrease in the concentration of miR-21 after the TSST.

However, the significant correlation between concentrations of miR-21 with the concentration of alpha-amylase indicates an association of miR-21 with the sympathetic activation. Interestingly, a direct interaction of miR-21 with adrenaline as an important transmitter for the sympathetic activation of the autonomous nervous system has already been described in recent literature^32^.

In the study of Honda *et al*., the concentrations of miR-16, miR-20b, miR-26b and miR-29a assessed in whole blood were increased in healthy male students who perceived chronic mental stress due to an upcoming examination as a psychological stressor^22^. Previously, miR-16 and miR-26b had been reported to be associated with psychological stress responses or psychiatric disorders^38^. Likewise, miR-16 has been associated with depressive disorder, especially being integrated in the regulation of the de novo expression of serotonin transporter (SERT)^39^. Furthermore, the miR-16 family appears to be associated with several different types of cancer, and it is suggested to be possibly useful as biomarker in whole blood, serum, plasma, urine or tissue, for those cancer diseases^40^.

While in the study of Honda *et al*. stress-responsive changes in miR-16 concentration in whole blood were significant, we could detect changes in the concentration of salivary miR-16, which were close to the significance level (p_Friedman_ = 0.059). The concentration of miR-16 significantly correlates with the VAS, indicating the increase of miR-16 to be associated to the subjective stress-perception. Interestingly, a possible integration of miR-16 in higher cortical functions – such as coping mechanisms of psychological stress – was already described in current literature; Hunsberger *et al*. reported an association of miR-16 with stress-associated psychiatric disorders^38^. Furthermore the expression of miR-16 in noradrenergic cells has been described to reduce the expression of SERT in these cells as an important factor in the serotonin-metabolism and the pathophysiology of depressive and other psychiatric disorders^39^. Thus, it might that a stress-related increase of miR-16 could affect remodeling processes in association with perceived psychological stress.

miR-20b expression levels have been reported to be altered in the prefrontal cortex of depressed suicide subjects^41^. Even though changes in the concentration of salivary miR-20b were significant, significant correlations were only found with miR-29a.

miR-26b has been described to be associated with neurogenesis^42^ and to be decreased in patients with hepatocellular carcinoma^43^. Although an association of miR-26b-concentrations with sympathetic activation might be suggested by its significant correlation with the concentration of salivary alpha-amylase in our study, such an association could not be found in current literature. However, this assumption is corroborated by the significant correlation between miR-26b and miR-21, a miRNA also highly assumed to be associated with the sympathetic activation^32^.

The miR-29 family has been reported to be associated with several different psychopathologies^24,44,45^. Interestingly, the expression level of miR-29c - as another member of the miR-29 family - was increased in the blood of healthy humans in response to the TSST and is therefore suggested to possibly function as a biomarker in blood for acute psychological stress response^24^. Further, miR-29a showed significant correlations with all of the reliably detectable miRNAs in our study, although changes in its own concentration were not significant. This might be due to the small number of participants or to imprecise detections of miR-29a. Nevertheless, these positive correlations suggest that the expression miR-29a is linked to the expression of the other miRNAs investigated in our study.

miR-134 has previously been described to be increased in the amygdala of rats in an acute psychological (restraint) stress model, showing also a trend to decrease in chronic psychological stress^25^. miR-134 is known to target several regulators of alternative splicing, it appears to regulate the dendritic spine development in the brain^46^ and appears to be associated with psychiatric disorders such as schizophrenia^38^. Furthermore, one of its targets has been reported to be causally involved in controlling mechanisms of oxidative stress and inflammation^25,47^.

Even though the increase of miR-134 directly after the TSST only showed a trend, close to significance, the post-hoc effect size calculation suggests larger effects in an increased sample size. Therefore, miR-134 could still be seen as an interesting and promising factor for further investigations of its integration in stress-associated processes – even in human saliva, not only in brain tissue.

There are several weaknesses of our study: (1) It was only explorative in character, based on the calculated sample size of n = 24, with estimated effect sizes and without precedents. (2) Our explicit selection of 11miRNAs was based on current findings about stress-associated miRNAs in general, without available knowledge about their presence in human saliva. This may be a reason why we could not detect 5 of the 11 miRNAs tested. (3) Focusing on such an explicit selection of 11 miRNAs we used a specific quantitative measuring method investigating those 11 miRNAs only. Regarding the limited knowledge on stress-associated miRNAs in human saliva, a high-throughput approach such as miRNA next-generation sequencing^48^ might have been preferable to provide a stress-associated miRNA-profile in human saliva. In the setting of this exploratory study design, such methods were not available due to financial and technical limitations.

There are also decisive strengths to our study. We demonstrate significant changes in the concentrations of salivary miRNAs as well as their correlations with established stress parameters responding classically to the TSST. Hence, we also demonstrate that the TSST is an appropriate tool for investigating salivary miRNAs as epigenetic biomarkers in acute psychological stress reactions. To our knowledge this is the first study to provide an indication for the usability of salivary epigenetic biomarkers for the investigation of acute psychological stress.

Furthermore, we show that the quantitative Polymerase Chain Reaction (qPCR) method is an appropriate method for investigating salivary miRNAs, even in conditions of low salivary miRNA-concentrations.

## Conclusions

Regarding our aims and specific research questions, our results demonstrate that, (1) stress-associated changes in the concentrations of some miRNAs in human saliva can be found responding to the TSST, either significantly or in a trend close to significance; (2) the TSST is an appropriate tool for the investigation of such miRNAs in a setting of acute psychological stress and (3) several correlations between miRNAs and established stress-associated parameters can be found, indicating specific associations of certain miRNAs with various aspects of the stress response. Namely, miR-21 and miR-26b appear to be associated with the sympathetic activation.

In this way our exploratory study provides a first experimental ground for on the study of stress-associated salivary miRNAs in acute psychological stress. From this starting point, multiple research routes could be followed to explore the detailed roles, sources and functions of miRNAs in human saliva as a novel and non-invasive medium in stress research.

Based on these results, we plan a consecutive three-armed randomized controlled study to investigate the effects of two established but competitive non-pharmacological stress interventions and a control group without intervention in stress-exposed healthy participants. For this study, both, miRNA-qPCR and miRNA next-generation sequencing are foreseen as technical methods. Results of such and similar studies might contribute to elucidating the roles of salivary miRNAs as non-invasive biomarkers for acute psychological stress reactions.

## Methods and Materials

### Participants/Subjects

24 healthy participants (12 female, 12 male) between 20 and 35 years old were recruited, among a population of University-students. Due to the fact of sex differences in stress responsibility^49^, which apply mainly to the first half of the menstrual cycle^50^ and to suspected sex differences in miRNA-expression^51^, we chose equal numbers of female and male participants. Moreover, the female participants did not take any hormonal contraception and had to be in the second half of their menstrual cycle at the date of participation. All participants fulfilled the criteria of in- and exclusion, i.e., they were in good physical and psychological health, had no history of psychiatric diseases, were non-smoking, taking no drugs, alcohol or medication, and did not perform any type of meditation or relaxation-exercises regularly (more than once a week). To minimize interference with circadian variations of cortisol levels, all tests were carried out between 3 and 5 p.m.

All experiments were conducted in accordance with the Declaration of Helsinki. The study was approved by the ethical committee of Witten/Herdecke University, Germany (number 96/2015) and registered in the German Register for Clinical Studies DRKS (Deutsches Register für klinische Studien), which is linked to the WHO-Register (Registration-ID: DRKS00010134). The participants were informed about the study aims and procedures, particularly their participation in a psychosocial stress test, and the timetable of the experiment was explained. In order to prevent reduced stress reactions due to prior mental adaption to the expected task, participants were not informed about the specific details of the TSST. Written informed consent of each participant was obtained before implementation of the experiment, and the participants were debriefed at the end of the experiment, receiving full information about the test and a symbolic reward of 10 Euros for their participation.

### Experimental stress test

As a standardized acute psychosocial stress test we applied the TSST^6^, a reliable, widely applied and internationally established standardized acute psychosocial stress test. We mainly followed the TSST-protocol by Birkett^52^, with modification of the time-points of sample collection (Fig. 1).

The protocol included 3 periods, a waiting period of 30 min, a test period (stress test) of 20 min, and a recovery period of 60 min (Fig. 1). During the waiting and recovery period, the participants were located on their own in a separate room with comfortable seating in a quiet atmosphere. The participants were asked not to use any electronic media.

For the TSST, the participants were separately brought to the actual testing room, the social laboratory, where they had to prepare (10 min) and then deliver (5 min) an oral presentation about the topic why they would be the best candidates for a personally ideal job-offer. After the speech the participants had to perform a mental arithmetic task (5 min), sequentially subtracting 13 from 1,022. If they made a mistake, they were told to start again from 1,022. The procedure took place in front of ‘experts’- a panel of 2 persons who followed a strict protocol. They wore white lab coats, exhibited unemotional neutrality, avoided any oral or mimic feedback, and just adverted if there was still time remaining or if a mistake was made during the mental arithmetic task. Furthermore, a video camera and a microphone were installed, which seemed to be running, but did not record at all.

### Assessment of stress parameters

Aside from the miRNAs as epigenetic parameters, subjectively perceived stress as well as generally accepted physiological and biochemical stress parameters were assessed.

#### Subjectively perceived stress

To assess subjectively perceived stress, a VAS ranging from 0 (feeling no stress at all) to 100 (feeling maximally stressed) was used at 9 time-points; before (T1 + T2), during (T3 − T5) and after (T6 − T9) the stress elicitation task^30,53^ (Fig. 1).

#### Physiological stress parameter

As physiological stress parameters, we analyzed the HR and HRV at 9 time-points analogously to the VAS-measurements T1 − T9 (Fig. 1). The ECG was continuously recorded throughout the procedure. Time markers were set at the beginning and at the end of each period by the participants to enable proper identification of the 9 analysis periods. Five minutes at the end of each period were analyzed with respect to HRV parameters (Fig. 1).

The Holter device’s internal sampling rate of the ECG was 4,096 Hz. Hence, the internally detected times of the R-peaks had a precision <1 ms. The times of the R-peaks and the ECG at a sampling rate of 256 Hz were saved on a memory card. The internally detected times of R-peaks were subsequently visually checked and corrected in case of false detections due to e.g. artifacts (<1% of all detected R-peaks). Subsequently, the RR interval series was calculated as the temporal distance between successive R-peaks. This series served as the basis for the HRV analysis.

Here, we calculated the average RR interval, SDNN and RMSSD to assess HRV in the time domain^54^.

#### Biochemical parameters

For the measurement of the biochemical and epigenetic parameters, saliva (5 ml each – including a sample part for miRNA-analysis) was collected at four time-points (T1, T5, T8, T9) (Fig. 1). After collection, saliva samples were directly stored at −80 °C. Storage, laboratory preparation and measurements were made using Eppendorf® (Hamburg, Germany) sterile and PCR-clean tubes.

The concentration of salivary cortisol as a marker for the activation of HPA-axis^7^ was measured by using a commercial (solid phase) enzyme-linked immunosorbent assay (ELISA) by IBL International® (Hamburg, Germany). The antigen-antibody reaction was stained using a test specific solution and a photometric measurement of the optical density (OD) was performed quantifying the reaction.

Activation of salivary alpha-amylase as a surrogate marker for sympathetic activation^8^ was measured, using a commercial (liquid phase) enzymatic assay by IBL International® (Hamburg, Germany). The measurements of salivary alpha-amylase activation levels allow conclusions to be drawn about the expression level of salivary alpha-amylase. The OD was measured at two time points (3 minutes and 8 minutes) after initiating the enzyme-substrate reaction, calculating ΔOD- values (OD_8 min – OD_3 min) afterwards.

For the measurement of both, salivary cortisol and amylase, test internal standards were provided in pairs, quality controls and samples were provided in triplets. Using 96-well plates, 24 saliva samples (all 4 samples of 6 participants) were provided at once. Mean OD-values (for salivary cortisol), respectively mean ΔOD- values (for salivary amylase) and standard deviations were calculated for further assessment. To ensure the quality control of the test procedures, the standard curve and internal quality controls were analyzed. As such quality controls were appropriate, no repeats of the test procedures had to be performed.

#### Epigenetic parameters

11 microRNAs (miR-10a; -16; -20b; -21; -26b; -29a; -126; -134; -144; -144* -183) in the exosomal fraction of saliva were investigated as epigenetic parameters.

miRNA-Isolation: The protocol used is mainly based on the protocol described by Michael *et al*.^15^. Modifications were established in our laboratory in a pre-test period.

After storage at −80 °C the samples were thawed in a water bath at +4 °C and aliquoted at 2 ml each. They were then centrifuged firstly at 1.500 g for 10 min, secondly at 17.500 g for 15 min, both times transferring the supernatant to a fresh tube, removing unwanted debris and cell fragments. Following these steps, the samples were then centrifuged at 160.000 g for 1 hour at +4 °C in a Sorvall M120GX centrifuge. Afterwards, the supernatant was removed and the pellet containing salivary exosomes was transferred to another tube, using 1 ml of TRIzol ® (ThermoFisher Massachusetts, USA), following the addition of 200 µl of chloroform for the extraction of RNAs from the exosomes. After mixing for 30 seconds, resting for 3 min at room temperature and centrifugation at 17.500 g for 15 min, the aqueous layer (RNA-phase) was transferred to another tube. 500 µl of Isopropanol was added and the RNA was precipitated at −20 °C for 20 min. The samples were then centrifuged at 17.500 g for 15 min, the supernatant was discarded and the pellet was washed with 1 ml of 75% Ethanol. After mixing and another centrifugation at 11.000 g for 5 min, the supernatant was discarded and the pellet was dried at room temperature for 20 min. For solubilization, 25 µl of RNAse-free water was added and samples were incubated at +60 °C for 15 min.

Reverse Transcription-PCR (RT-PCR): After miRNA-isolation from salivary exosomes, 12 µl of RNA-solution was used to generate a specific single-strand complementary DNA (cDNA) by reverse transcription polymerase chain reaction (RT-PCR) using the “miScript II RT-Kit” (Qiagen, Hilden, Germany). After RT-PCR all samples were stored at −20 °C or immediately used for real-time quantitative PCR.

Real-time quantitative-PCR (qPCR): The cDNA-solution from the previous step was used for real-time quantitative PCR using the “miScript SYBR Green PCR-Kit” (Qiagen, Hilden, Germany). For all miRNAs, triple measurements were made, calculating the mean and later ΔCT-values using ‘snU6’^55^ as an internal control. Cut off values of triplet-measurements were set at CT-values >37, differences in between the triplets of more than +/−1 CT and differences of melting curves +/− 0,5.

Lower ΔCT-values indicate higher miRNA concentrations in the sample.

### Statistical analysis

The objective of this exploratory study was the assessment of stress-related miRNAs changes detected in the exosomal fraction of human saliva. Based on estimated effect of d = 0.6, a power of 80%, and a significance level of 0.05 a total number of 24 subjects were calculated to detect significant differences across the TSST procedure. Because of the low number of participants, the distribution of the parameters was quantified by the median and the interquartile range (25%-/75%-percentile; IQR).

Consistently, non-parametric statistical procedures were used. Friedman test for repeated measures was calculated to assess changes of the parameters at the times T1, T5, T8 and T9 (miRNAs, saliva cortisol, saliva alpha amylase) or at the times T1 to T9 (VAS, mean RR interval, SDNN, RMSSD). In case of missing values (VAS, miR-26b and miR-134), the Skillings-Mack test was used as a replacement for the Friedman test to take advantage of all available data^56^. If the Friedman test (or the Skillings-Mack test) showed significant changes of a parameter, pairwise differences between different times were checked post-hoc including adjustment for multiple comparisons^57^.

To obtain an impression of the overall strength and direction of changes in miRNAs, and to impart confidence whether the a priori estimated effect sizes were justified, effect sizes η^2^ were calculated based on the results of the Friedman or Skillings-Mack test using the Minsize 2 computer program^58^. For reasons of comparability and interpretability η^2^ effect sizes were then transferred into Cohen’s d using the algorithms giving in Cohen^59^.

Additionally, to assess relationships between the parameters, correlations were calculated using the Spearman rank correlation procedure.

In all statistical calculations a p < 0.05 was considered significant. With respect to the changes of miRNAs we also report on results with a tendency towards significance, i.e. p < 0.10. Thus, miRNAs are also identified which likely show significant changes with a slightly increased number of cases.

### Data availability

The datasets generated and analyzed in the current study are available from the corresponding author on reasonable request.

### Ethics approval and consent to participate

All experiments were conducted in accordance with the Declaration of Helsinki. The study was approved by the ethical committee of Witten/Herdecke University, Germany (number 96/2015) and registered in the German Register for Clinical Studies DRKS (Deutsches Register für klinische Studien), which is linked to the WHO-Register (Registration-ID: DRKS00010134).

### Availability of data and materials

The datasets used and analyzed during the current study are available from the corresponding author on reasonable request.
